# When endoscopic full-thickness resection goes wrong: strategy for resolution

**DOI:** 10.1055/a-2851-8344

**Published:** 2026-04-30

**Authors:** Maria Moreno-Sanchez, Álvaro Martinez-Alcalá

**Affiliations:** 1 Gastroenterology 145708 University Hospital Infanta LeonorMadridSpain

We present the case of a 75-year-old man with a long-standing history of a laterally spreading tumor, granular homogeneous type located in the distal rectum. The original 60-mm lesion was treated by piecemeal endoscopic mucosal resection (EMR) in 2010, with multiple adenomatous recurrences subsequently managed endoscopically. At follow-up colonoscopy in 2025, an 18-mm adenomatous recurrence could not be treated with standard EMR because of significant fibrosis.

**Fig. 1:**
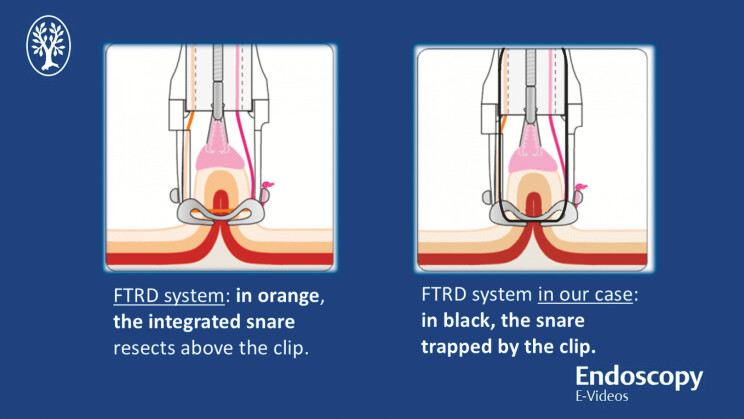
A full-thickness resection device: on the left, there is a normal functioning system. Represented in orange, there is the integrated snare that resects above the over-the-scope (OTS) clip. On the right, there is a diagram of what happened in our case: represented in black, there is the snare trapped underneath the OTS clip.

Given the lesion size and morphology, a hybrid EMR–endoscopic full-thickness resection (EFTR) approach was chosen. The less fibrotic lateral component was initially resected by EMR, reducing the residual fibrotic area to less than 15 mm and making the lesion suitable for EFTR in terms of both the size and morphology 1 . After successful positioning and activation of the system, with release of the over-the-scope (OTS) clip and snare resection ( Video 1 ), it became apparent that the integrated snare had been inadvertently trapped underneath the OTS clip ( Fig. 1 ). Nonetheless, the lesion was successfully resected and sent for histopathological examination.

EFTR: once the lesion is inside the cap, the OTS-clip is released. The snare in the FTRD gets trapped by the OTS-clip. This is solved by a “single-port laparoscopy” technique. EFTR, endoscopic full-thickness resection; FTRD, full-thickness resection device; OTS, over-the-scope.Video 1

**Fig. 2:**
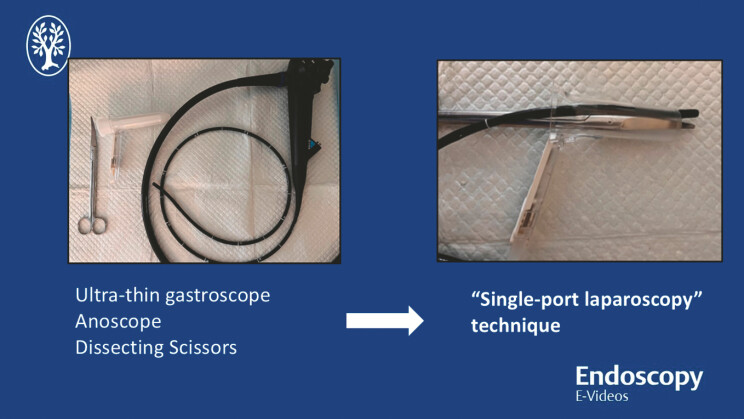
A “single-port laparoscopy” technique: through an anoscope, we inserted an ultrathin gastroscope and surgical dissection scissors (Metzenbaum), to cut the trapped snare under direct vision.

To address this complication, the colonoscope was first freed by cutting the distal end of the trapped diathermy snare with pliers. The FTRD kit was then removed and the rectum re-intubated. An additional diathermy snare was introduced through the free end of the trapped snare in an attempt to release it, without success. Finally, a “single-port laparoscopy” technique was applied ( Fig. 2 ): through an anoscope, an ultrathin gastroscope and surgical dissection scissors (Metzenbaum) were inserted, allowing the trapped snare to be cut under direct vision.

Endoscopic follow-up demonstrated granulation tissue without recurrence.

This case highlights a rare but relevant EFTR-related complication, possibly related to device malfunction or human error. With the expanding use of rectal EFTR, a flexible, cross-disciplinary approach (incorporating instruments and concepts from other specialties) may represent an effective rescue strategy for complex endoscopic adverse events.

Endoscopy_UCTN_Code_CPL_1AJ_2AD_3AF
